# Spiraling elliptic Hermite-Gaussian solitons in nonlocal nonlinear media without anisotropy

**DOI:** 10.1038/s41598-017-03669-x

**Published:** 2017-06-12

**Authors:** Guo Liang, Zhiping Dai

**Affiliations:** 1 School of Electrical & Electronic Engineering, Shangqiu Normal University, Shangqiu, 476000 China; 2 College of Physics and Electronic Engineering, Hengyang Normal University, Hengyang, 421002 China

## Abstract

We introduce a kind of the spiraling elliptic Hermite-Gaussian solitons in nonlocal nonlinear media without anisotropy, which carries the orbital angular momentum and can rotate in the transverse. The *n*–th mode of the spiraling elliptic Hermite-Gaussian solitons has *n* holes nested in the elliptic profile. The analytical spiraling elliptic Hermite-Gaussian solitons solutions are obtained based on the variational approach, which agree well with the numerical simulations. It is found that the critical power and the critical angular velocity for the spiraling elliptic Hermite-Gaussian solitons are the same as the counterpart of the ground mode.

## Introduction

The nonlinear propagation of optical beams with orbital angular momentum (OAM) has been discussed in recent years. The spiraling beams carrying the OAM can exert forces and torques on the microparticles, which make them rotate^1^. The technologies associated with OAM, including spatial light modulators and hologram design, have found their own applications ranging from optical tweezers to microscopy^2^. Spiraling solitons^3^ with the OAM are usually associated with optical vortices^4, 5^ and the ring-shaped beams^6^. Meanwhile, the vortex-free beams with nonzero OAM are well known in linear media, such as an elliptically shaped beam focused by a tilted cylindrical lens^7^. It was discussed that the OAM has two contributions to the dynamics of elliptic beams in nonlinear self-focusing media^8^. First, it effectively strengthens the diffraction against self-focusing and can suppress collapse in Kerr media. Second, it preserves the elliptic profile of stably rotating solitons in optical media with collapse-free nonlinearities. Spiraling elliptic solitons have been found to exist in the media with saturable nonlinearity^8^ and nonlocal nonlinearity^9^. It was claimed that OAM can result in the effective anisotropic diffraction for the spiraling elliptic beams^9^. And the deviations from the critical OAM can make the spiraling elliptic beams breathe^10^. The decrease (increase) of the OAM can make the spiraling elliptic breathers converge (diffract). Introducing linear anisotropy in the nonlinear media, the OAM will not be conserved. Depending on the linear anisotropy of the media, two kinds of evolution behaviors for the dynamic breathers, rotations and molecule-like librations were predicated analytically and confirmed in numerical simulations^11^.

In this paper, we discuss a kind of spiraling elliptic Hermite-Gaussian solitons in nonlocal nonlinear media, the *n*–th mode of which has *n* holes nested in the elliptic profile. In particular, the fundamental mode is the spiraling elliptic solitons^8–12^. By using the variational approach, we obtained the approximate analytical solutions, which agree with the numerical simulations well. We find the critical angular velocity of such a soliton depends on the initial parameters but does not depend on its order, which has potential applications in the controlling of the optical beams.

## Model

The propagation of optical beams in nonlocal cubic nonlinear media can be modeled by the following nonlocal nonlinear Schrödinger equation (NNLSE)^13–15^

1 $$i\frac{\partial \psi }{\partial z}+\frac{1}{2}{\nabla }_{\perp }^{2}\psi +{\rm{\Delta }}n\psi =\mathrm{0,}$$

where ${\nabla }_{\perp }^{2}=\frac{{\partial }^{2}}{\partial {x}^{2}}+\frac{{\partial }^{2}}{\partial {y}^{2}}$ is the transverse Laplacian operator, *ψ*(*x*, *y*, *z*) is the complex amplitude envelope, ${\rm{\Delta }}n=\iint R(x-x^{\prime} ,y-y^{\prime} ){|\psi (x^{\prime} ,y^{\prime} )|}^{2}dx^{\prime} dy^{\prime}$ is the light-induced nonlinear refractive index, *z* is the longitudinal coordinate, *x* and *y* are the transverse coordinates, and *R* is the normalized symmetrical real spatial response function of the media such that ∫∫*R*(*x*, *y*)*dxdy* = 1. In the strongly nonlocal nonlinear (SNN) media, we only need keep the first two terms of the expansion of Δ*n*. Then, the NNLSE is simplified to the Snyder-Mitchell mode (SMM)^13^

2 $$i\frac{\partial \psi }{\partial z}+\frac{1}{2}{\nabla }_{\perp }^{2}\psi -\frac{1}{2}\gamma {P}_{0}({x}^{2}+{y}^{2})\psi =\mathrm{0,}$$

where $\gamma =-\frac{1}{2}{\partial }_{x}^{2}R(x,y){|}_{x=0,y=0}=-\frac{1}{2}{\partial }_{y}^{2}R(x,y){|}_{x=\mathrm{0,}y=0}$, *P*_0_ = ∫∫ |*ψ*(**r**′)|^2^d^2^ **r**′ is the input optical power, and **r**′ is the transverse coordinate vector with **r**′ = *x*′**e**_*x*_ + *y*′**e**_*y*_. Although the SMM (2) is a phenomenological model, it can keep the main features of the SNN media. For example, the theoretical predictions by the Snyder-Mitchell model^13^, such as the accessible solitons and the attraction of spatial solitons, have been observed in experiments in the nematic liquid crystal^16–18^ and the lead glass^19^. It is worth mentioning that the fractional Fourier transform existing the SNN media was predicted by the SMM^20^, which was also observed in the lead glass.

**Figure 1:**
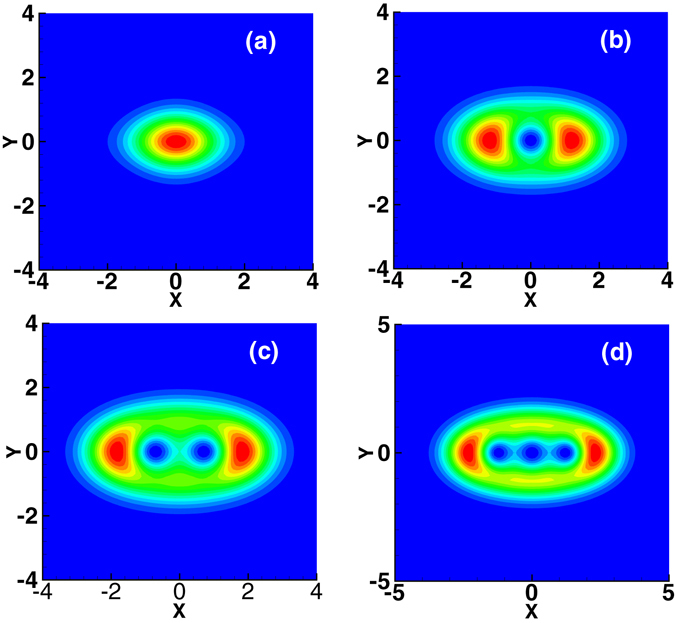
Profiles of lowest order spiraling elliptic Hermite-Gaussian beam. (**a**) Fundamental mode with *n* = 0; (**b**) first-order mode with *n* = 1; (**c**) second-order mode with *n* = 2; (**d**) third-order mode with *n* = 3. The parameters of all figures are *b* = 1.2 and *c* = 0.8 in Eq. (3).

Optical beam carrying the orbital angular momentum (OAM) has been investigated in the nonlocal nonlinear media modeled by Eq. (1) ^9, 10, 21^. Hermite soliton clusters in nonlocal nonlinear media have been introduced by Buccoliero *et al*.^22^. The spiraling elliptic Hermite-Gaussian beam carrying the OAM is introduced as follows

3 $$\psi (X,Y,Z)={A}_{n}(Z){H}_{n}(X+iY)\exp [-\frac{{X}^{2}}{2{b}^{2}(Z)}-\frac{{Y}^{2}}{2{c}^{2}(Z)}]\exp (i\varphi )$$

where *H*_*n*_ is the *n*–order Hermite polynomials, *b*(*z*) and *c*(*z*) are the semi-axes of the elliptic beam, *A*_*n*_ is a parameter in connection with the amplitude of the optical beam, and *ϕ* = *B*(*z*)*X* ^2^ + Θ(*z*)*XY* + *Q*(*z*)*Y* ^2^ + *ϑ*(*z*) is the phase. The *n*–order spiraling elliptic Hermite-Gaussian beam has the elliptic profile, and has *n* holes aligned along the direction of the principal axis of ellipse. Figure 1 shows the lowest order spiraling elliptic Hermite-Gaussian beam as examples. It should be noted that the expression (3) of the elliptical beam is in the rotating coordinate system *XYZ*, where *X* = *x* cos *β*(*z*) + *y* sin *β*(*z*), *Y* = −*x* sin *β*(*z*) + *y* cos *β*(*z*), *Z* = *z*. And in the static coordinate system *xyz*, the optical beam (3) will rotate carrying the OAM during propagation, the angular velocity of which can be obtained as *ω* = *dβ*/*dz*. There exists a significant difference between the spiraling elliptic Hermite-Gaussian beam (3) and the complex-variable-function-Gaussian beams introduced in ref. 21, which is, the phase contains an additional cross term Θ*XY* in the former case. The fundamental mode of (3) with *n* = 0 corresponds to the spiraling elliptic solitons discussed in our recent work^9^. The optical power and the OAM can be obtained by inserting Eq. (3) into the following two formulas *P*_0_ = ∫∫ |*ψ*(*x*′, *y*′)|^2^ *dx*′*dy*′ and ${M}_{0}={\rm{Im}}\iint {\psi }^{\ast }(x\frac{\partial \psi }{\partial y}-y\frac{\partial \psi }{\partial x})dxdy$. For the three lowest orders, as examples, the optical power and the OAM have been obtained inTable 1. And the similar procedure can be employed for other higher-order modes. As can be seen from Table 1, for the fundamental mode of the spiraling elliptic Hermite-Gaussian beams, the OAM results from the cross term Θ*XY* on its phase, but for other high-order mode both the cross term Θ*XY* and the Hermite polynomials *H*_*n*_(*X* + *iY*) contribute to the OAM.

**Table 1:** Parameters of the spiraling elliptic Hermite-Gaussian beam for different orders of *n*.

	*P*_0_	*σ* ≡ *M*_0_/*P*_0_
*n* = 0	*πA* ^2^ *bc*	(*b* ^2^ − *c* ^2^)Θ/2
*n* = 1	2*πA* ^2^ *bc*(*b* ^2^ + *c* ^2^)	[2 + 3(*b* ^2^ − *c* ^2^)Θ]/2
*n* = 2	4*πA* ^2^ *bc*[3(*b* ^4^ + *c* ^4^) − 2*b* ^2^ *c* ^2^ − 2(*b* ^2^ − *c* ^2^)]	*C*1(*b*, *c*) + *C*2(*b*, *c*)Θ^*^

*$${C}_{1}(b,c)=\frac{\mathrm{23(}{b}^{4}+{c}^{4})+2{b}^{2}{c}^{2}-{b}^{2}+{c}^{2}}{\mathrm{3(}{b}^{4}+{c}^{4})+2{b}^{2}{c}^{2}-\mathrm{2(}{b}^{2}-{c}^{2})+1},$$

$${C}_{2}(b,c)=\frac{15{b}^{6}+3{b}^{4}({c}^{2}-2)+{b}^{2}(1+4{c}^{2}-3{c}^{4})-{c}^{2}(1+6{c}^{2}+15{c}^{4})}{\mathrm{6(}{b}^{4}+{c}^{4})+4{b}^{2}{c}^{2}-\mathrm{4(}{b}^{2}-{c}^{2})+2}$$.

## Variational solution

Based on the variational approach^23^, Eq. (2) can be expressed as an Euler-Lagrange equation corresponding to the variational principle

4 $$\delta {\int }_{-\infty }^{\infty }{\int }_{-\infty }^{\infty }{\int }_{-\infty }^{\infty }l(\psi ,{\psi }^{\ast },{\psi }_{x},{\psi }_{x}^{\ast },{\psi }_{y},{\psi }_{y}^{\ast },{\psi }_{z},{\psi }_{z}^{\ast })dxdydz=0$$

with the Lagrangian density given by ref. 24

5 $$l=\frac{i}{2}({\psi }^{\ast }\frac{\partial \psi }{\partial z}-\psi \frac{\partial {\psi }^{\ast }}{\partial z})-h,$$

where *h* is the Hamiltonian density expressed as

6 $$h=\frac{1}{2}({|\frac{\partial \psi }{\partial x}|}^{2}+{|\frac{\partial \psi }{\partial y}|}^{2}+\frac{1}{2}\gamma {P}_{0}({x}^{2}+{y}^{2}){|\psi |}^{2})\mathrm{.}$$

Inserting the trial solution of spiraling elliptic Hermite-Gaussian beam (3) into the Lagrangian $L={\int }_{-\infty }^{\infty }{\int }_{-\infty }^{\infty }ldxdy$, and then, following the standard procedures of the variational approach^23^, we can obtain that

7 $$d{P}_{0}/dz=d{M}_{0}/dz=0,$$

8 $$B=\frac{\mathrm{(3}{b}^{4}+6{b}^{2}{c}^{2}+{c}^{4})b^{\prime} -2{b}^{3}cc^{\prime} }{2b\mathrm{(3}{b}^{4}+4{b}^{2}{c}^{2}+{c}^{4})},$$

9 $$Q=\frac{({b}^{4}+6{b}^{2}{c}^{2}+3{c}^{4})c^{\prime} -2b{c}^{3}b^{\prime} }{2c({b}^{4}+4{b}^{2}{c}^{2}+3{c}^{4})},$$

10 $${\rm{\Theta }}=\frac{\mathrm{2(}\sigma -\mathrm{1)}}{\mathrm{3(}{b}^{2}-{c}^{2})},$$

where the primes indicate derivatives with respect to the variable *z*. Thus it can be found that the power and the OAM of the system are conservative. In the following analytical calculations, we take the first-order mode of spiraling elliptic Hermite-Gaussian beam (3) as an example. And the similar calculations can be applied to other higher modes. The angular velocity can be obtained as

11 $$\omega =\frac{d\beta }{dz}=\frac{2}{\mathrm{3(}{b}^{2}+{c}^{2})}+\frac{\mathrm{(3}{b}^{4}+2{b}^{2}{c}^{2}+3{c}^{4}){\rm{\Theta }}}{\mathrm{3(}{b}^{4}-{c}^{4})},$$

which reveals that the angular velocity *ω* is closely related to the OAM. Substituting the trial solution (3) into the Hamiltonian density (6) and carrying out integration $H={\int }_{-\infty }^{\infty }{\int }_{-\infty }^{\infty }hdxdy$, we obtain the Hamiltonian of the system, which is the function of *b*, *c*, *B*, *Q* and Θ. As did in our previous work^9^, by the substitution of Eqs (8), (9) and (10), the Hamiltonian is expressed by *H*(*b*′, *c*′, *b*.*c*), which is the summation of the generalized kinetic energy *T* and the generalized potential energy *V*. The generalized kinetic energy *T* is a quadratic function of the generalized velocity *b*′ and *c*′. If we assume that *T* = 0, i.e. *b*′ = *c*′ = 0, we can obtain the potential energy *V* = *H*(*b*, *c*) as follows

12 $$\begin{array}{rcl}V & = & \frac{{P}_{0}}{72}[\frac{\mathrm{18(}{b}^{2}+{c}^{2})}{{b}^{2}{c}^{2}}-{P}_{0}\gamma ({b}^{2}-3{c}^{2})+\frac{\mathrm{8(}\sigma -{\mathrm{1)}}^{2}}{{(b-c)}^{2}}\\  &  & +\frac{\mathrm{8(}\sigma -{\mathrm{1)}}^{2}}{{(b+c)}^{2}}+\frac{\mathrm{8(}\sigma +{\mathrm{2)}}^{2}+36{P}_{0}\gamma {b}^{4}}{{b}^{2}+{c}^{2}}]\mathrm{.}\end{array}$$

Solitons can be found as the extrema of the potential *V*(*b*, *c*). By setting ∂*V*/∂*b* = 0 and ∂*V*/∂*c* = 0, we can obtain the critical power and the critical OAM

13 $${P}_{c}=\frac{{({\rho }^{2}+\mathrm{1)}}^{2}}{2\gamma {b}^{4}},$$

14 $${\sigma }_{c}=\frac{3{\rho }^{4}-2{\rho }^{2}+3}{4{\rho }^{2}},$$

respectively, and *M*_*c*_ = *P*_*c*_ *σ*_*c*_, where *ρ* = *b*/*c* represents the ellipticity of the elliptic beam. Substitution of the critical power (13) and the critical OAM (14) into the expression of the angular velocity (11) yields

15 $${\omega }_{c}=\frac{{b}^{2}+{c}^{2}}{2{b}^{2}{c}^{2}},$$

which shows that the spiraling elliptic Hermite-Gaussian solitons make constant-angular rotations. Then the period of rotation can be obtained as *T* = 2*πb* ^2^ *c* ^2^/(*b* ^2^ + *c* ^2^).

For high-order mode of the spiraling elliptic Hermite-Gaussian beams, the critical power and the critical OAM can be calculated by the same process. It is found that the critical power and the critical angular velocity are the same as the counterpart of the ground mode, i.e. Eqs (13) and (15) respectively. While, it is different for the other critical parameters, for example, when *w*_*m*_ = 20, *b* = 1.2, *c* = 0.8, we obtain *σ*_*c*_ = 2.76925 and Θ_*c*_ = 0.434028 for the second-order mode of the spiraling elliptic Hermite-Gaussian solitons.

## Numerical simulation

Here we take the Gaussian function as the spatial nonlocal response function^25, 26^, i.e.

16 $$R(x,y)=\frac{1}{2\pi {w}_{m}^{2}}\exp (-\frac{{x}^{2}+{y}^{2}}{2{w}_{m}^{2}}),$$

then the parameter *γ* in the SMM (2) is obtained $\gamma =\mathrm{1/(}\pi {w}_{m}^{4})$. Although the Gaussian response function is phenomenological, which does not exist in any physical system, it can be employed to obtain the analytic solution of the NNLSE (1). In addition, for any reasonable response function the physical properties do not depend strongly on its shape. The generic properties of different types of response functions have been studied by Wyller *et al*. in terms of modulational instability^27^.

**Figure 2:**
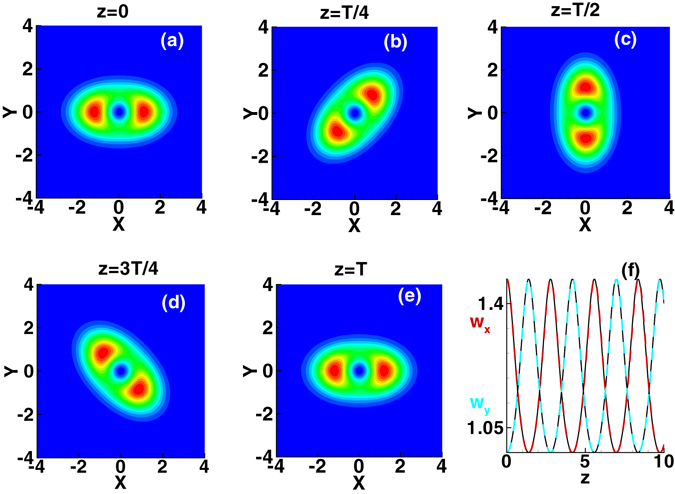
Evolution of the first-order mode of the spiraling elliptic Hermite-Gaussian solitons. Profiles are plotted at different propagation distances: (**a**) *z* = 0, (**b**) *z* = *T*/4, (**c**) *z* = *T*/2, (**d**) *z* = 3*T*/4, and (**e**) *z* = *T*(=2.78). The beam width *w*_*x*_ and *w*_*y*_ in the *x* and *y* directions are plotted in (**f**), where solid black lines and dashed color lines denote the variational solution and numerical simulations respectively. The parameters are *b* = 1.2, *c* = 0.8, and *w*_*m*_ = 20.

**Figure 3:**
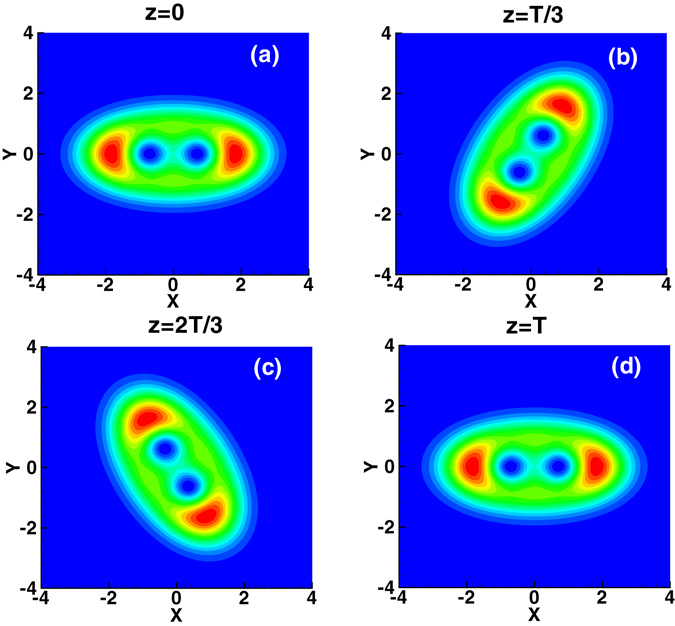
Evolution of the second-order mode of the spiraling elliptic Hermite-Gaussian solitons.

**Figure 4:**
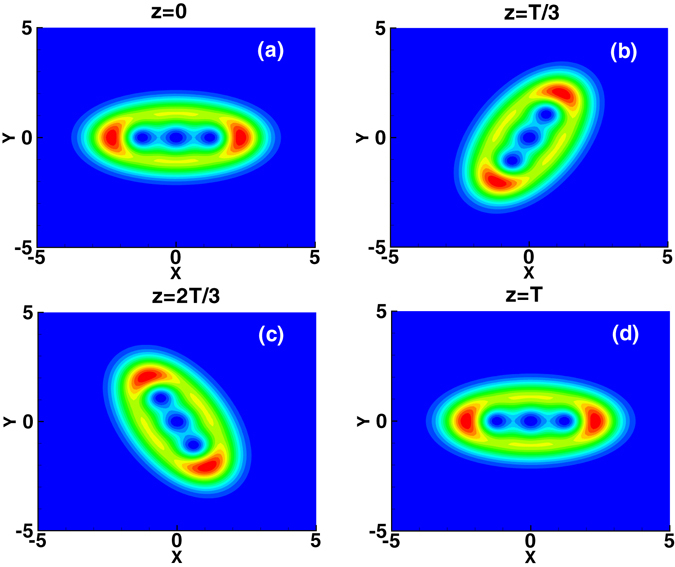
Evolution of the third-order mode of the spiraling elliptic Hermite-Gaussian solitons.

The method of numerical simulation used here is the split-step Fourier method^28^ using the variational solution (3) as the input. The evolution of the first-order mode of the spiraling elliptic Hermite-Gaussian solitons is shown in Fig. 2, where the parameters are *b* = 1.2, *c* = 0.8, and *w*_*m*_ = 20. The second-order-moment beam widths based on the variational solution are obtained as ${w}_{x}={[\mathrm{(2/3)(}{b}^{-2}{\cos }^{2}{\omega }_{c}z+{c}^{-2}{\sin }^{2}{\omega }_{c}z)]}^{-\mathrm{1/2}}$ and ${w}_{y}={[\mathrm{(2}/3)({c}^{-2}{\cos }^{2}{\omega }_{c}z+{b}^{-2}{\sin }^{2}{\omega }_{c}z)]}^{-\mathrm{1/2}}$ along the *x* and *y* directions, which agrees with the numerical simulations very well as shown in Fig. 2(f). It reveals that the variational solution, including any order spiraling elliptic Hermite-Gaussian solitons is valid for the SNN media, as shown in Figs 3 and 4.

**Figure 5:**
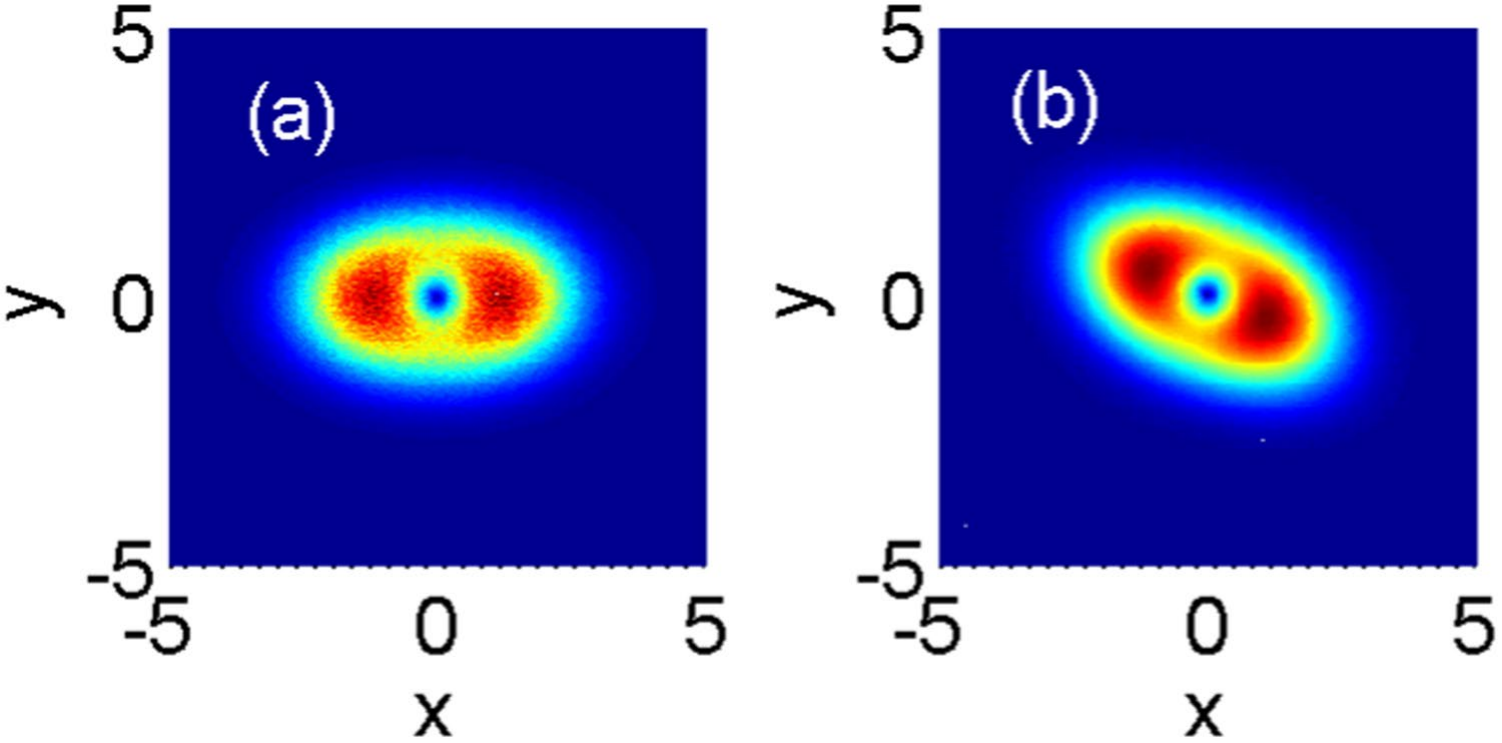
First-order mode of the spiraling elliptic Hermite-Gaussian solitons [i.e. the soliton profiles in Fig. 1(b)] with 20% random noises (**a**), and the profile at the propagation distance *z* = 20 (**b**).

To address the stability of the spiraling elliptic Hermite-Gaussian solitons, we performed numerical simulations of Eq. (2) by employing the initial condition as [1 + *εf*(*x*, *y*)]*ψ*(*x*, *y*), where *ψ*(*x*, *y*), *f*(*x*, *y*) are spiraling elliptic Hermite-Gaussian solitons and the random function with maximum amplitude less than 1, and *ε* denotes the perturbation parameter. Figure 5 presents the nonlinear propagations of the first-order mode of the spiraling elliptic Hermite-Gaussian solitons with 20% random noises, where we can find the profiles remain invariant up to *z* = 20. Other high-order modes of the spiraling elliptic Hermite-Gaussian solitons exhibit similar dynamics with added random noises. Of course, the solitons can propagate much farther with random noises than we did in the simulation, which in fact shows the spiraling elliptic Hermite-Gaussian solitons are stable in the nonlocal nonlinear media.

## Conclusion

We have introduced a kind of spiraling elliptic Hermite-Gaussian solitons in nonlocal nonlinear media, which carries the the orbital angular momentum and has the elliptic profile with *n* holes aligning along the direction of the principal axis of ellipse for the *n*–th order mode. Based on the variational approach, we obtained the approximate analytical solutions, which agree with the numerical simulations well. It was found that the critical power and the critical angular velocity are the same as the counterpart of the ground mode, irrespective of the order *n*.
